# Frequency masking drives species-specific temporal avoidance strategies in boreal songbirds

**DOI:** 10.1093/beheco/araf154

**Published:** 2025-12-22

**Authors:** Agata Staniewicz, Adrianna Muszyńska, Emilia Sokołowska, Michał Budka

**Affiliations:** Department of Behavioural Ecology, Adam Mickiewicz University in Poznań, Uniwersytetu Poznańskiego 6, Poznań 61-614, Poland; Bat Conservation Trust, Studio 15 Cloisters House, 8 Battersea Park Road, London SW8 4BG, United Kingdom; Department of Behavioural Ecology, Adam Mickiewicz University in Poznań, Uniwersytetu Poznańskiego 6, Poznań 61-614, Poland; Department of Behavioural Ecology, Adam Mickiewicz University in Poznań, Uniwersytetu Poznańskiego 6, Poznań 61-614, Poland; Department of Behavioural Ecology, Adam Mickiewicz University in Poznań, Uniwersytetu Poznańskiego 6, Poznań 61-614, Poland

**Keywords:** song avoidance, signal space, acoustic interference, song overlap

## Abstract

Interference in acoustic signal transmission can impair communication, and many vocalizing species use various strategies to avoid signal masking. Past studies have focused primarily on the effect of anthropogenic noise and overlap in sound frequency range, leaving a gap in our understanding of how an individual's vocal signal structure affects other animals in its acoustic community. Using playback experiments we tested whether 5 species of European boreal songbirds adjust their singing behavior to avoid overlap with 3 novel acoustic intruders from sub-Saharan Africa, each with a different song structure customized to each study species: (i) continuous songs of narrow, partially-masking frequency based on the study species’ peak frequency, (ii) noncontinuous songs of broad, fully-masking frequency and (iii) continuous songs of broad, fully-masking frequency. All species showed evidence of temporal song avoidance only with competitors whose songs were broad-spectrum and completely overlapped in frequency range. The species differed in their behavioral response, either modifying the song rate, song duration, or the timing of their songs. These differences in behavior may be related to limitations imposed by the species-specific information encoded in different song parameters, and allow for predicting the consequences of changes in acoustic community structure.

## Teaser

Many animals change their vocal behavior to avoid overlapping sounds and ensure clear communication. We studied five boreal forest songbird species and found that they adjust their singing when faced with new bird songs that completely mask their frequency range. The birds either altered how often they sang, how long their songs lasted, or when they sang, highlighting how different species in the forest community adapt their communication strategies to their acoustic environment.

## Introduction

An acoustic community comprises all animals vocalizing in the same environment. Each species in the community occupies its own specific region of the acoustic space, determined by the frequency range, time and amplitude of its vocalizations (Nelson and Marler 1990). When 2 individuals produce sounds which overlap in both frequency and time, the resulting acoustic interference can alter or completely mask their signals (Dooling 1982; Klump 1996). This limits detection and in the case of broadcasting signals can lead to fewer mating opportunities, lowering the individual's reproductive success (Bradbury and Vehrencamp 2011). As the frequency range of a species' signal is correlated with its body size (Podos 2001; Fletcher 2004) and phylogeny (Bolanos-Sittler et al. 2021; Mikula et al. 2021), many coexisting species will occupy the same frequency range and overlap in their vocalizations. Consequently, according to the acoustic partitioning hypothesis (Hödl 1977), species should employ competition avoidance strategies to reduce signal interference.

Previous studies focusing on acoustic community level have identified different strategies of acoustic space partitioning aimed at mitigating the competition between species vocalizing in the same frequency range at the same time. These include modifying the timing or duration of the individual's signals to fit in the intervals between the vocalizations of other species (Brumm 2006; Bleach et al. 2015; Lenske and La 2014; Herrick et al. 2018), increasing the amplitude to mitigate the masking effect of a competing vocalization (Brumm 2004; Kunc and Schmidt 2021), or adjusting the frequency of the signal by shifting above or below the frequency of the interfering sound (Lopez et al. 1988; Verzijden et al. 2010; Goodwin and Podos 2013).

Birds are a particularly vocal taxonomic group, with many species using songs for mate attraction and territory defense (Catchpole and Slater 2008). Many species sing at the same time, with the most prominent examples being the morning peak of vocal activity known as the dawn chorus. Different interspecific competition mitigation strategies have been demonstrated in bird acoustic communities, and include examples of song overlap avoidance by partitioning on either temporal scale (Cody and Brown 1969; Popp et al. 1985; Lenske and La 2014; Wilson et al. 2016; Hart et al. 2021), spectral scale (Kirschel et al. 2009; Krishnan and Tamma 2016), or both (Luther 2009). However, not all studies provide support for the acoustic partitioning hypothesis. Similarly singing birds were found to aggregate, rather than separate, in space and time in both tropical (Tobias et al. 2014) and temperate forest communities (Staniewicz et al. 2023). Planque and Slabbekoorn (2008) and Berman et al. (2024) found little evidence for song overlapping avoidance, except at the frequency range most commonly used by birds or in case of complete frequency overlap with cicadas.

Finally, although temporal partitioning has been documented as a strategy for avoiding acoustic competition in both individual bird species (Lenske and La, 2014; Wilson et al. 2016) and entire communities (Cody and Brown 1969; Popp et al. 1985; Hart et al. 2021), relatively little attention has been paid to interspecific differences in these strategies within the same community. Similarly, the role of song structure and frequency overlap in shaping competitive interactions has been underexplored. For instance, species that share the same acoustic community but differ in song structure may respond differently to acoustic interference: in sub-Saharan Africa green-backed camaroptera which have broad-spectrum songs may increase their song rate, while scaly-breasted illadopsis with narrow-spectrum songs may reduce theirs in response to the same masking sound (Budka et al. 2023; Staniewicz et al. 2024). Such contrasting responses in song rate could be driven by different factors such as differences in how information is encoded in the songs, differences in both the structure and total frequency range of their own songs, as well as those of the interfering species. These factors are likely critical in determining a species' susceptibility to acoustic masking, yet they have received limited attention in research to date.

Here we tested the temporal adjustment response of 5 species of European boreal forest songbirds with different song frequency and structure to 3 unfamiliar acoustic competitors with different song structure and thus different levels of temporal and spectral overlap. Compared with tropical and temperate regions, boreal forests have a lower bird species diversity and density (Mönkkönon 1994). These results in less complex acoustic environment compared with areas with higher species richness and more vocalizing taxa. Such conditions offer the opportunity to closely examine the effects of competition between pairs of species in natural conditions. We used playback to simulate the appearance of different acoustic intruders: (i) one with continuous songs of narrow frequency, which could overlap the target species in time but mask only a portion of its song's on the frequency spectrum; (ii) one with noncontinuous songs of broad frequency, which could mask the target species on the frequency spectrum but not completely overlap in time; and (iii) one with continuous songs of broad frequency which could overlap with target species in time and completely mask its song's on the frequency spectrum. We expected the birds to alter their behavior in response to the different intruders, and adjust their song rate and singing pattern to avoid masking. We predicted that they would sing fewer songs during the playback of the acoustic competitor which completely masked them in both temporal and frequency domain, than during the playbacks of partially-masking competitors or periods of silence. We also predicted that the birds would avoid starting their songs or temporally overlapping with the songs of the acoustic competitor that could completely mask them in both frequency and time domain.

## Methods

### Study site

Ekopark Käringbereget is located in the central boreal zone (Ahti et al. 1968) of northern Sweden (N 64°5′22″ E 18°37′38″) and is a managed forest landscape composed of a mixture of production and conservation forest sites. The area encompasses 14,000 ha dominated by Scots pine and Norway spruce, with some parts of birch and aspen (Ekström et al. 2021). We conducted our study between 22 May and 7 June 2023, with the mean temperature 9 °C (ranging between 2 and 20 °C), and the day length increasing from 19 h 3 min (22 May: sunrise 02:13, sunset 21:15) to 20 h 31 min (7 June: sunrise 01:31, sunset 22:02).

### Study species

**Figure 1:**
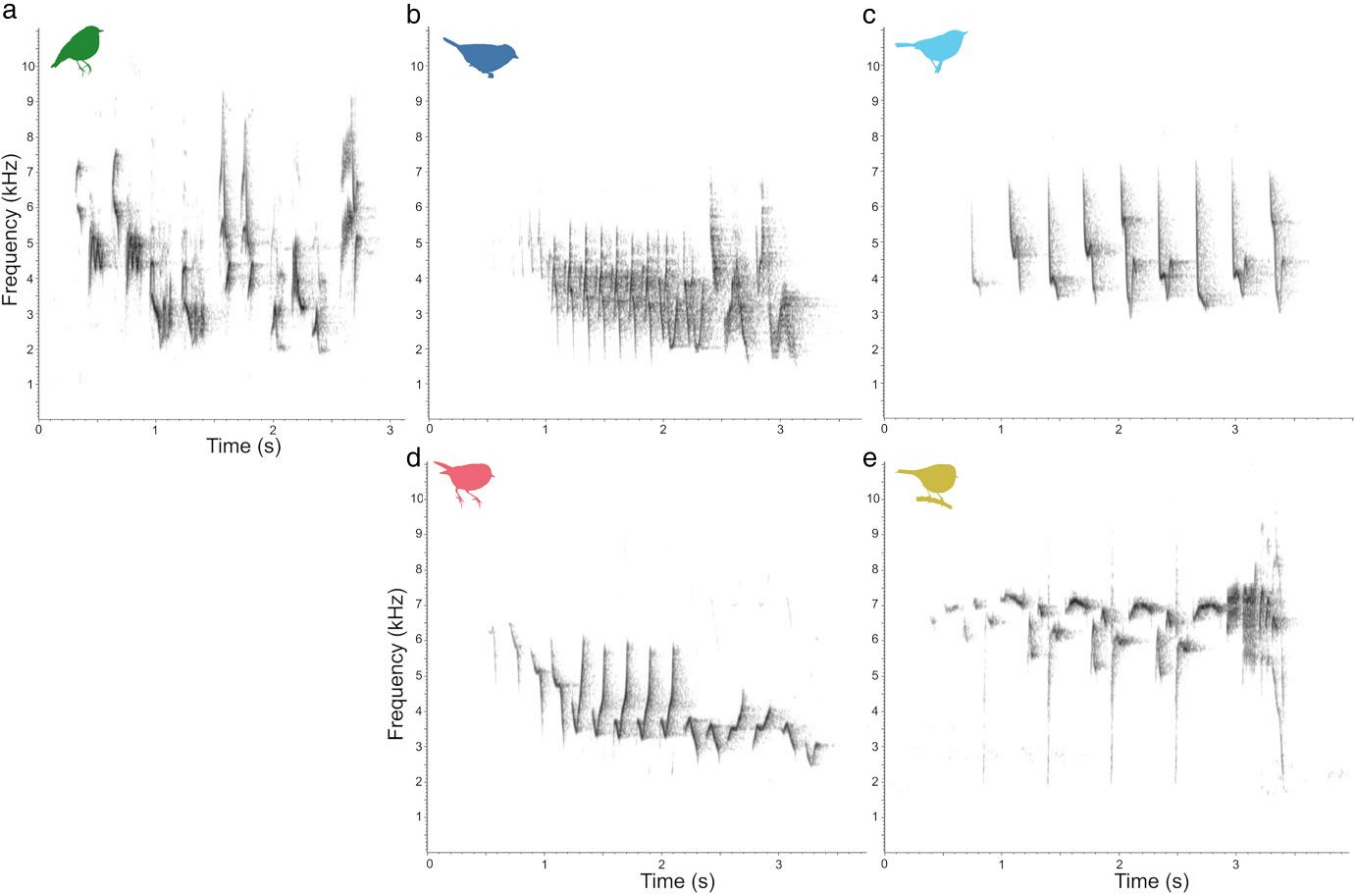
Spectrograms of natural songs of the 5 study species: a) European pied flycatcher (*Ficedula hypoleuca*); b) common chaffinch (*Fringilla coelebs*), c) common chiffchaff (*Phylloscopus collybita*); d) willow warbler (*Phylloscopus trochilus*); e) goldcrest (*Regulus regulus*). Sampling rate 48 kHz; FFT size 1024; Hanning window, overlap 75%.

We focused on 5 species breeding at the study site: European pied flycatcher (*Ficedula hypoleuca*), common chaffinch (*Fringilla coelebs*), common chiffchaff (*Phylloscopus collybita*), willow warbler (*Phylloscopus trochilus*), and goldcrest (*Regulus regulus*). These species were all commonly encountered at the study site, produced species-specific, stereotyped songs and occupied different parts of the frequency spectrum. The pied flycatcher is a migratory species, wintering in West Africa and breeding in Sweden from late April to end of June (Taylor and Christie 2020). It is a cavity nester and prefers forest habitats (Taylor and Christie 2020). Males sing near the nest site, with the song composed of 5 to 15 notes of alternating pitch (Fig. 1a). The common chaffinch is partially migratory, with birds from Scandinavia wintering in western Europe, and breeding from March to mid-July. The species is widespread in forests, glades, heaths and agricultural areas (Clement et al. 2023). Males produce a characteristic song composed of a series of descending notes followed by an accelerated finish (Fig. 1b). The common chiffchaff (*Phylloscopus collybita*) is migratory, wintering in sub-Saharan Africa. It breeds from April to early August, preferring mixed and deciduous forest, especially alder and willow (Clement et al. 2020). Males often sing from tall birch trees near glades, producing a characteristic song of varying length repeating 2 alternating notes (Fig. 1c). The willow warbler (*Phylloscopus trochilus*) also migrates to sub-Saharan Africa. It breeds between April and July in deciduous and mixed forests particularly with birch (Clement 2020). Its song is composed of a series of alternating descending notes (Fig. 1d). The goldcrest (*Regulus regulus*) is a short-range migrant wintering in central and southern Europe. The species prefers coniferous boreal forests and breeds from end of March (Martens and Päckert 2020). Males produce a song composed of several high-pitched notes followed by a variable finish (Fig. 1e).

### Playback preparation

We collected reference recordings for the 5 study species in Ekopark Käringberget between 18 and 31 May 2021. We recorded naturally singing birds using a Sennheiser ME67 shotgun directional microphone with a K6 powering module connected to Marantz PMD661 digital recorder (wav file, 48 kHz sampling rate with 16-bit accuracy). We measured between 5% and 95% of the total energy distributed in the songs to determine the mean frequency range for each species (Table 1) using the automated measurements for each selected song in Raven Pro v1.6.1 software (https://ravensoundsoftware.com). To account for variation between songs and individuals we included the standard deviation (SD) in reported minimum and maximum frequency for each species. We calculated the mean and SD of the minimum frequency of all the songs measured for the species, and subtracted the SD from the mean to obtain the minimum frequency for the species. We calculated the mean and SD of the maximum frequency of all the songs measured for the species, and added the SD to the mean to obtain the maximum frequency for the species. We used this information to prepare the playback recordings of acoustic intruders.

**Table 1:** Frequency ranges containing 90% of the energy of the signal, and the peak frequency of the songs of the 5 study species, measured from the reference recordings.

Species	Individuals (*n*)	Songs (*n*)	Min frequency (mean-SD) at 5% (kHz)	Max frequency (mean + SD) at 95% (kHz)	Peak frequency (mean ± SD) (kHz)
**Goldcrest**	6	43	5.6	7.5	6.6 ± 0.4
**Common chiffchaff**	6	51	3.4	6.1	4.4 ± 0.5
**European pied flycatcher**	9	80	2.8	6.1	4.2 ± 0.5
**Willow warbler**	7	57	2.8	5.9	4.3 ± 0.4
**Common chaffinch**	6	49	2.4	4.9	3.5 ± 0.4

Sampling rate 48 kHz; FFT size 1024; Hanning window, overlap 75%.

**Figure 2:**
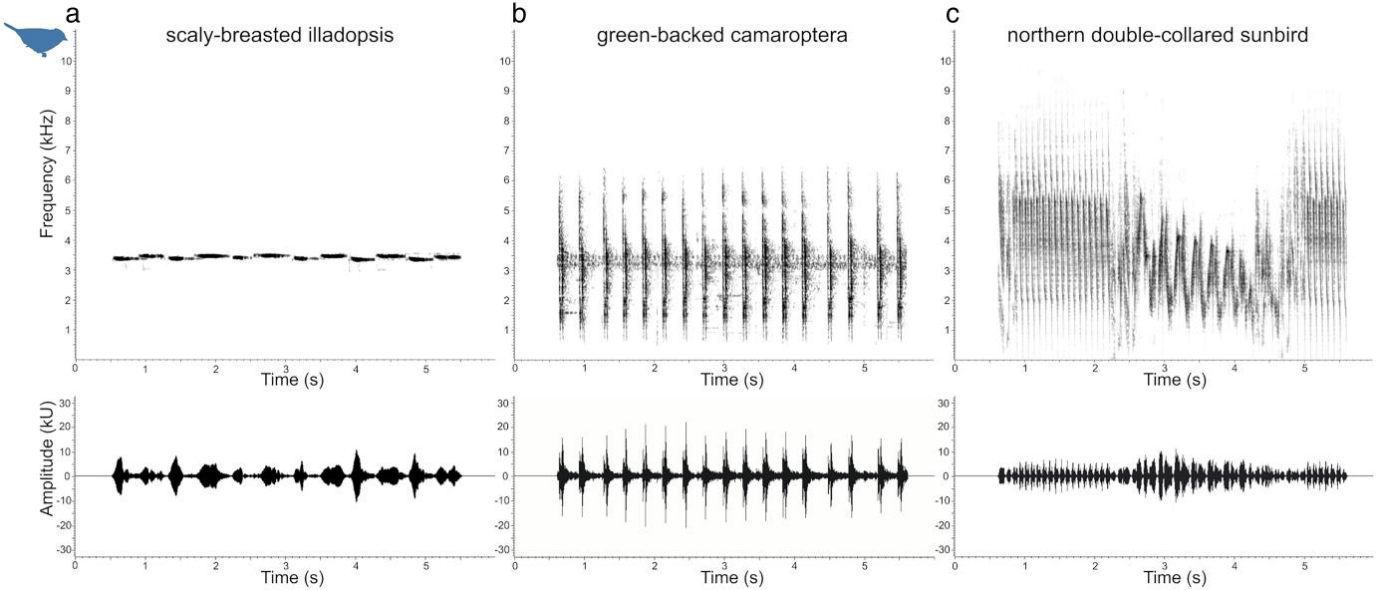
Example spectrograms and oscillograms of modified 5-s acoustic competitor songs with frequency adjusted for playback to the common chaffinch; a) scaly-breasted illadopsis (*Illadopsis albipectus*); b) green-backed camaroptera (*Camaroptera brachyura*); c) northern double-collared sunbird (*Cinnyris reichenowi*). Sampling rate 44.1 kHz; FFT size 1024; Hanning window, overlap 75%.

For acoustic intruders we selected 3 Afrotropical species with different song structures: (i) scaly-breasted illadopsis (*Illadopsis albipectus*) with a narrow-frequency spectrum range song consisting of 3 notes sang 1 after the other without pause (Fig. 2a); (ii) green-backed camaroptera (*Camaroptera brachyura*) with a broad-spectrum frequency song consisting of a series of individual sharp notes with pauses between them (Fig. 2b); (iii) northern double-collared sunbird (*Cinnyris reichenowi*) producing a broad-spectrum song with no pauses between notes (Fig. 2c). All 3 species are sedentary and inhabit the forests of sub-Saharan Africa. While their range may overlap with overwintering pied flycatchers, common chiffchaffs and willow warblers, which may have provided some degree of familiarity with the sounds, the acoustic intruder playback songs were modified for each of the study species.

We selected 2 to 3 songs of 6 individuals of each intruder species from good-quality recordings collected during previous work in Kibale National Park, Uganda (camaroptera and illadopsis, June 2022) and Bamenda Highlands, Cameroon (sunbird, November-December 2014–2016). We used Avisoft SasLab Pro 5.2.12 software (https://www.avisoft.com) to downsample the recordings to 44.1 kHz with 16-bit accuracy, normalize the amplitude to 95% and remove offset. To prepare playback recordings tailored to individual study species and simulate acoustic interference in both spectral and temporal domain, we shifted the songs of the acoustic intruders to match the peak frequency of each of the target species, ensuring that the broad-frequency songs of green-backed camaroptera and sunbird fully overlapped with the target species song frequency range (Table 1). We set the duration of each song to 5 s by increasing the number of syllables where necessary (Fig. 2), to create playback treatments of alternating equal periods of song and silence. Each playback sound sample lasted 40 min, containing 4 identical 10-min sessions of 4 150-s treatments, each of 5-s continuous songs alternating with 5-s silent gaps of one of the novel acoustic competitors (camaroptera, CB; illadopsis, IA; sunbird, CR) or silence as a control. Thus, during each 10-min session, the target bird would be exposed to all 3 acoustic competitors as well as silence. In every playback sequence for a given study species we used a different song from each of the novel acoustic competitors. We focused our playback treatments only on sounds overlapping in frequency with the study species' songs as we wanted to explore the effects of the overlapping sound's structure and the level of temporal and spectral overlap on the species' response. Past studies (Budka et al. 2023; Staniewicz et al. 2024) have demonstrated that birds avoid overlapping temporally with interfering sounds that overlap their song frequency range, but not with sounds which do not overlap their song frequency range.

### Experimental procedures

We carried out the experiments between 22 May and 7 June 2023, between 0255 and 0945 h. We searched for singing birds from forest roads and trails and upon locating a bird we observed it for 10–15 min to determine its core singing territory. We only selected birds with territories where no conspecifics could be heard singing in the vicinity, and ensured that all the tested birds were out of earshot range from each other. We positioned 2 speakers (JBL Charge 5) approximately 20 m from each other on tree branches approximately 4 m above ground, near the center of the area where the bird was singing the most. The speakers were connected via Bluetooth to a smartphone. We waited to ensure the bird was singing before commencing the playback. Each bird was presented with 4 10-min sessions of 4 experimental treatments (the 3 acoustic competitor species and silence as a control, broadcast in random order), each lasting 150 s and played at 86 ± 1 dB at 1 m. Each 150 s session was played from only 1 speaker, and we alternated the active speaker between the sessions to reduce habituation.

Two observers recorded the experiment, 1 using Rode NTG8 shotgun microphone (frequency response 40 Hz–20 kHz; 20.0 dB; 1 V/Pa (97.5 mV at 94 dB SPL) ± 2 dB at 1 kHz) connected to a Sound Devices MixPre 3 recorder, the other using an Olympus LS-12 handheld recorder with built-in stereo microphones. Each observer noted when the bird was singing, as well as whether the bird has moved away from the playback range (defined as >50 m away from the nearest speaker) and its position relative to the speakers. Additionally, to record any songs that may be inaudible to the observers, we placed 1 Song Meter Mini (Wildlife Acoustics) recorder with a single built-in omnidirectional microphone (signal to noise ratio 78 dB at 1 kHz; sensitivity + 6 dB ± 4 dB at the 18 dB gain setting used) within the singing range but away from the observers.

We tested 12 individuals of each species (60 birds in total). In case of the pied flycatcher, chiffchaff and goldcrest, each of the 12 tested individuals was presented with a different combination of the acoustic competitor song samples in a random order of 150 s treatments (Table S1). Two of the 12 chaffinches and 2 of the 12 willow warblers tested were presented with the same playback treatment, while the remaining 10 individuals were presented with unique treatment combinations (see data in Supplementary Materials for details).

### Acoustic analysis

We recorded all experiments in wav format at sampling rate 48 kHz with 16-bit accuracy. We manually aligned all recordings from each tested bird and saved it as a single synchronized 3-channel wav file using Audacity v3.1.3 software (https://audacityteam.org). We then manually scanned each 3-channel recording using Raven Pro software and used the selection table tool to mark every song produced by the tested bird when it was within the range of the playback (total number of songs marked = 9,361). We measured the duration of each song and determined whether it overlapped in time with an acoustic competitor playback song by more than 50%. As all playback treatments involved equal 5 s intervals of playback song and silence providing equal possibilities of the song being produced during the silence or the interfering sound, we chose the >50% as a threshold for song overlap, which indicated the majority of the song—and the encoded information—would be masked by the playback song.

### Statistical analysis

All analyses were performed in R v4.2.1 (https://www.R-project.org). To test the effect of the acoustic competitor song structure type on the number of songs produced by the birds we used generalized linear mixed models (GLMMs; negative binomial distribution, log link) for each species using the glmmTMB package (Brooks et al. 2017). We calculated the odds ratios using emmeans v1.8.7 package (Length 2023). We set the number of songs produced by the bird during the 150 s playback treatment as the response variable, the treatment type (CB, CR, IA, or silence) and sequence of the 150 s playback phases within the 40 min experiment (from 1 to 16) as predictor variables (fixed effects), and the identity of the bird as a random effect.

To test the effect of the acoustic competitor song structure type on the duration of the songs produced by the birds we used similar GLMMs (negative binomial distribution, log link). We set the song duration (in seconds) as the response variable, the treatment type (CB, CR, IA, or silence) and sequence of the 150 s playbacks as predictor variables (fixed effects), and the identity of the bird as a random effect.

We compared the numbers of songs which overlapped with the playback for more than 50% of the song's duration between the 3 acoustic competitor playback treatments using logistic regression models for each species (glm function; binomial family; logit link). This included both the songs that started during the song of the acoustic competitor, as well as during the periods of silence. We set the response variable as binary (>50% overlap, <50% overlap) for each song, and the treatment type and sequence order of the 150 s playback treatments as the predictor variables. Pairwise comparisons of estimated marginal means were conducted using the emmeans package, with *P*-values adjusted using the Tukey method.

We prepared similar logistic regression models to test the association between the acoustic competitor song type and the likelihood that the bird would start its song while a competitor's song was being broadcast. This included both the songs that overlapped by more and by < 50% with the songs of the acoustic competitor. We set the response variable as binary (song began during playback or during silence) and the treatment type and sequence order of the 150 s playback treatments as the predictor variables. Pairwise comparisons of estimated marginal means were conducted using the emmeans package, with *P*-values adjusted using the Tukey method.

## Results

### Song number

**Figure 3:**
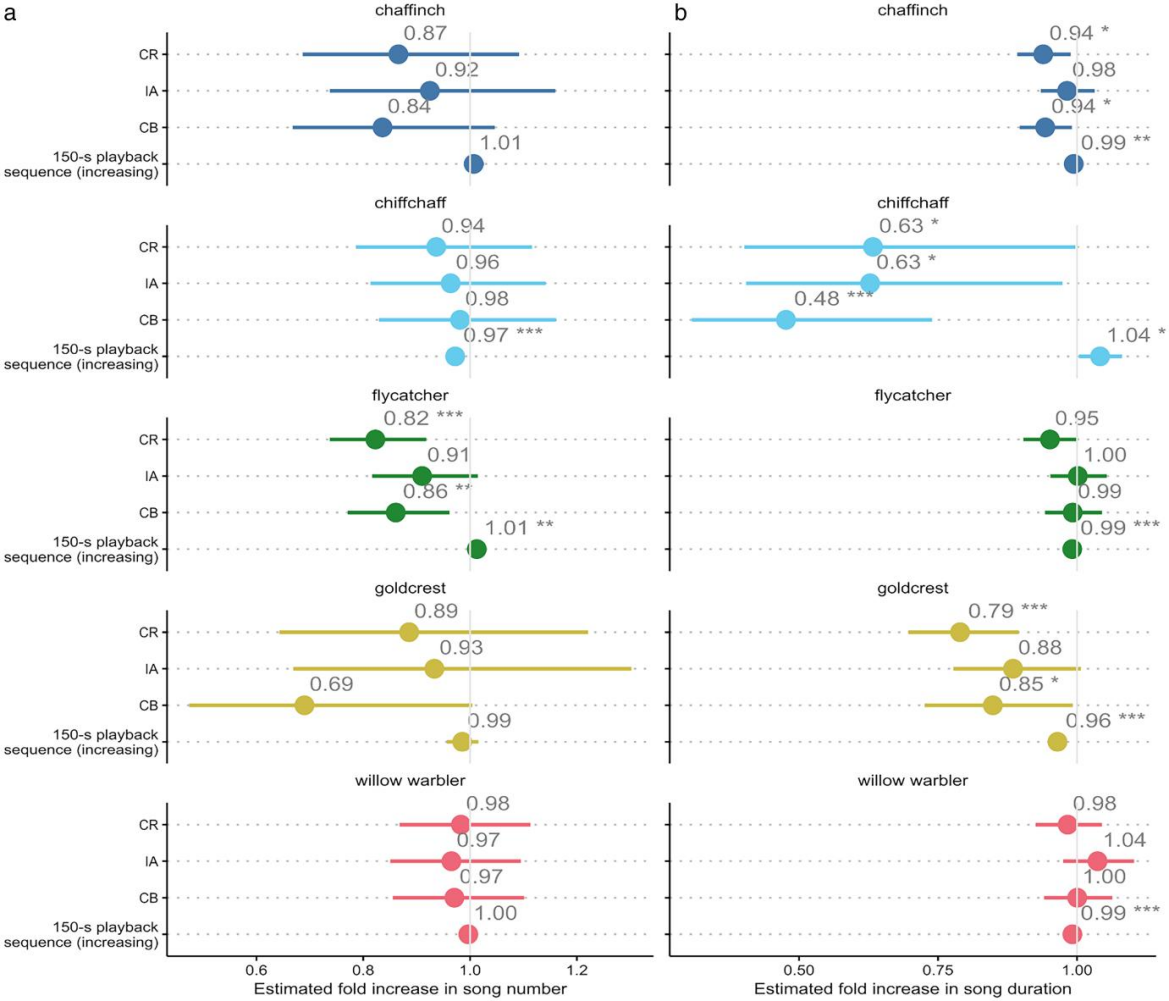
Odds ratios (ORs) of fixed effects estimates from the GLMMs predicting the difference in a) number of songs produced by the bird during a 150-s playback of acoustic competitor songs (sunbird, CR; illadopsis, IA; camaroptera, CB) compared with 150-s period of silence, with increasing experiment duration; b) duration of songs produced by the bird during a 150-s playback of acoustic competitor songs (sunbird, CR; illadopsis, IA; camaroptera, CB) compared with 150-s period of silence, with increasing experiment duration. *P < 0.05; **P < 0.01; ***P < 0.001. Bars represent 95% confidence intervals. Full model outputs are given in Tables 2 and 3.

The pied flycatcher produced significantly fewer songs during the 150 s playbacks of acoustic competitors masking its songs in both frequency and time (sunbird, CR; mean ± SD = 17.4 ± 5.46 songs; odds ratio, OR = 0.82; 95% confidence interval, CI = 0.74, 0.92; *P* < 0.001) and in frequency domain only (camaroptera, CB; mean ± SD = 18.3 ± 5.28 songs; OR = 0.86; 95% CI = 0.77, 0.96; *P* < 0.001) than during the 150 s periods of silence (mean ± SD = 21.1 ± 6.15). There was no significant difference between the numbers of songs the pied flycatchers produced during the playbacks of the narrow-frequency spectrum acoustic competitor (illadopsis, IA; mean ± SD = 19.2 ± 5.77 songs; *P* = 0.090) and periods of silence. However, there was a significant effect of experiment duration, with the birds producing significantly more songs during the later 150 s treatments (OR = 1.01, 95% CI = 1.00, 1.02; *P* = 0.004; Table 2; Fig. 3a).

**Table 2:** GLMMs examining the effects of acoustic competitor type (sunbird, CR; illadopsis, IA; camaroptera, CB) and the sequence of the 150 s playback treatments on the number of songs produced by 12 common chaffinch, 12 common chiffchaff, 12 European pied flycatchers, 12 willow warblers and 12 goldcrests.

	Coefficients	Estimate	SE	*Z*	*P*	Mean ± SD song number
**Common chaffinch**	Intercept	2.373	0.140	16.992	**<0**.**001**	12.1 ± 5.64
	CR	−0.144	0.118	−1.217	0.223	10.9 ± 5.88
	IA	−0.078	0.116	−0.677	0.499	11.7 ± 5.77
	CB	−0.179	0.115	−1.564	0.118	10.4 ± 5.80
	Sequence	0.007	0.007	0.711	0.477	
**Common chiffchaff**	Intercept	2.442	0.099	24.717	**<0**.**001**	10.5 ± 3.69
	CR	−0.065	0.089	−0.732	0.464	9.83 ± 4.6
	IA	−0.037	0.086	−0.428	0.668	10.0 ± 4.46
	CB	−0.019	0.086	−0.221	0.825	10.0 ± 4.45
	Sequence	−0.028	0.007	−3.961	**<0**.**001**	
**Pied flycatcher**	Intercept	2.933	0.066	44.26	**<0**.**001**	21.1 ± 6.15
	CR	−0.195	0.056	−3.49	**<0**.**001**	17.4 ± 5.46
	IA	−0.094	0.056	−1.70	0.090	19.2 ± 5.77
	CB	−0.150	0.056	−2.66	**0**.**008**	18.3 ± 5.28
	Sequence	0.013	0.004	2.88	**0**.**004**	
**Goldcrest**	Intercept	2.602	0.186	14.026	<0.001	13.4 ± 8.59
	CR	−0.121	0.164	−0.740	0.459	12.1 ± 6.58
	IA	−0.069	0.170	−0.407	0.684	12.4 ± 5.50
	CB	−0.371	0.192	−1.938	0.053	9.53 ± 6.13
	Sequence	−0.015	0.015	−0.954	0.340	
**Willow warbler**	Intercept	2.419	0.127	19.081	**<0**.**001**	12.2 ± 3.94
	CR	−0.017	0.063	−0.272	0.785	11.8 ± 3.62
	IA	−0.036	0.065	−0.550	0.582	11.9 ± 3.46
	CB	−0.030	0.064	−0.462	0.644	11.9 ± 3.89
	Sequence	−0.003	0.005	−0.631	0.528	

Mean and SD of the numbers of songs recorded during each playback treatment type.

In the common chiffchaff, there was no significant difference in the song rate between the periods of silence (mean ± SD = 10.5 ± 3.69 songs) and the playback treatments of 3 acoustic competitors (Table 2). However, the birds produced significantly fewer songs during the later 150 s treatments (OR = 0.97; 95% CI = 0.96, 0.98; *P* < 0.001; Table 2; Fig. 3a).

Common chaffinch, willow warblers and goldcrest showed no significant differences between the number of songs produced during the periods of silence and the playback treatments of 3 acoustic competitors (Table 2; Fig. 3a). There was also no significant effect of the experiment duration on the numbers of songs produced (Table 2; Fig. 3a).

### Song duration

The common chaffinch produced significantly shorter songs during the 150 s playbacks of acoustic competitors masking its songs in both frequency and time (sunbird, CR; mean ± SD = 2.40 ± 0.47 s; odds ratio, OR = 0.94; 95% confidence interval, CI = 0.89, 0.99; *P* = 0.017) and in frequency domain only (camaroptera, CB; mean ± SD = 2.38 ± 0.45 s; OR = 0.94; 95% CI = 0.90, 0.99; *P* = 0.021) than during the 150 s periods of silence (mean ± SD = 2.47 ± 0.46 s). There was no significant difference between the duration of songs produced during the playbacks of the narrow-frequency spectrum acoustic competitor (illadopsis, IA; mean ± SD = 2.42 ± 0.43 s; *P* = 0.473) and periods of silence. There was a significant effect of experiment duration, with the birds producing significantly shorter songs during the later 150 s treatments (OR = 0.99, 95% CI = 0.99, 0.99; *P* = 0.008; Table 3; Fig. 3b).

**Table 3:** GLMMs examining the effects of acoustic competitor type (sunbird, CR; illadopsis, IA; camaroptera, CB) and the sequence of the 150s playback treatments on the duration of songs produced by 12 common chaffinch, 12 common chiffchaff, 12 European pied flycatchers, 12 willow warblers and 12 goldcrests.

	Coefficients	Estimate	SE	*Z*	*P*	Mean ± SD song duration (s)
**Common chaffinch**	Intercept	2.493	0.089	27.882	**<0**.**001**	2.47 ± 0.46
	CR	−0.063	0.026	−2.397	**0**.**017**	2.40 ± 0.47
	IA	−0.018	0.025	−0.717	0.473	2.42 ± 0.43
	CB	−0.059	0.025	−2.312	**0**.**021**	2.38 ± 0.45
	Sequence	−0.006	0.002	−2.674	**0**.**008**	
**Common chiffchaff**	Intercept	5.745	0.452	12.712	**<0**.**001**	5.71 ± 2.99
	CR	−0.457	0.232	−1.972	**0**.**049**	5.22 ± 2.80
	IA	−0.465	0.224	−2.077	**0**.**038**	5.22 ± 3.04
	CB	−0.741	0.224	−3.306	**0**.**001**	5.22 ± 3.13
	Sequence	0.041	0.0190	2.142	**0**.**032**	
**Pied flycatcher**	Intercept	2.369	0.072	32.75	**<0**.**001**	2.28 ± 2.99
	CR	−0.050	0.026	−1.90	0.057	2.26 ± 0.63
	IA	0.001	0.026	0.05	0.956	2.29 ± 0.56
	CB	−0.008	0.026	−0.29	0.770	2.28 ± 0.61
	Sequence	−0.009	0.002	−4.13	**<0**.**001**	
**Goldcrest**	Intercept	3.133	0.124	25.264	**<0**.**001**	3.01 ± 0.79
	CR	−0.236	0.064	−3.697	**<0**.**001**	2.78 ± 0.84
	IA	−0.122	0.066	−1.850	0.064	2.88 ± 1.03
	CB	−0.164	0.080	−2.053	**0**.**040**	2.70 ± 0.73
	Sequence	−0.036	0.006	−5.618	**<0**.**001**	
**Willow warbler**	Intercept	3.194	0.135	23.627	**<0**.**001**	3.27 ± 0.63
	CR	−0.017	0.031	−0.545	0.586	3.26 ± 0.59
	IA	0.036	0.031	1.155	0.248	3.33 ± 0.64
	CB	0.001	0.031	0.012	0.991	3.31 ± 0.64
	Sequence	−0.008	0.002	−3.333	**<0**.**001**	

Mean and SD of the duration of songs recorded during each playback treatment type.

The common chiffchaff sang significantly shorter songs during all the playbacks of CR (mean ± SD = 5.22 ± 2.80 s; OR = 0.63; 95% CI = 0.40, 0.99; *P* = 0.049), IA (mean ± SD = 5.22 ± 3.04 s; OR = 0.63; 95% CI = 0.40, 0.97; *P* = 0.038) and CB (mean ± SD = 5.22 ± 3.13 s; OR = 0.48; 95% CI = 0.31, 0.74; *P* = 0.001) compared with the periods of silence (mean ± SD = 5.71 ± 2.99 s). However, the birds also produced significantly longer songs over the course of the experiment (OR = 1.04 95% CI = 1.01, 1.08; *P* = 0.032; Table 3; Fig. 3b).

In the pied flycatcher, there was no significant effect on the song duration during the periods of silence (mean ± SD = 2.28 ± 2.99 s) and the playback treatments of 3 acoustic competitors (Table 3). However, the birds produced significantly shorter songs during the later 150 s treatments (OR = 0.99; 95% CI = 0.98, 0.99; *P* < 0.001; Table 3; Fig. 3b).

Goldcrest songs were also significantly shorter during the 150 s playbacks of CR (mean ± SD = 2.78 ± 0.84 s; odds ratio, OR = 0.79; 95% confidence interval, CI = 0.70, 0.89; *P* = 0.040) and CB (mean ± SD = 2.70 ± 0.73 s; OR = 0.85; 95% CI = 0.73, 0.99; *P* = 0.001) than during the 150 s periods of silence (mean ± SD = 3.01 ± 0.79 s). There was no significant difference between the duration of songs produced during the playbacks of IA (mean ± SD = 2.88 ± 1.03 s; *P* = 0.064) and periods of silence. However, the birds produced significantly shorter songs during the later 150 s experimental treatments (OR = 0.96, 95% CI = 0.95, 0.98; *P* < 0.001; Table 3; Fig. 3b).

There was no significant effect on the song duration produced by the willow warbler during the periods of silence (mean ± SD = 3.27 ± 0.63 s) and the playback treatments of 3 acoustic competitors (Table 3). However, the birds produced significantly shorter songs during the later 150 s treatments (OR = 0.99; 95% CI = 0.98, 0.99; *P* < 0.001; Table 3; Fig. 3b).

### Song overlap

**Figure 4:**
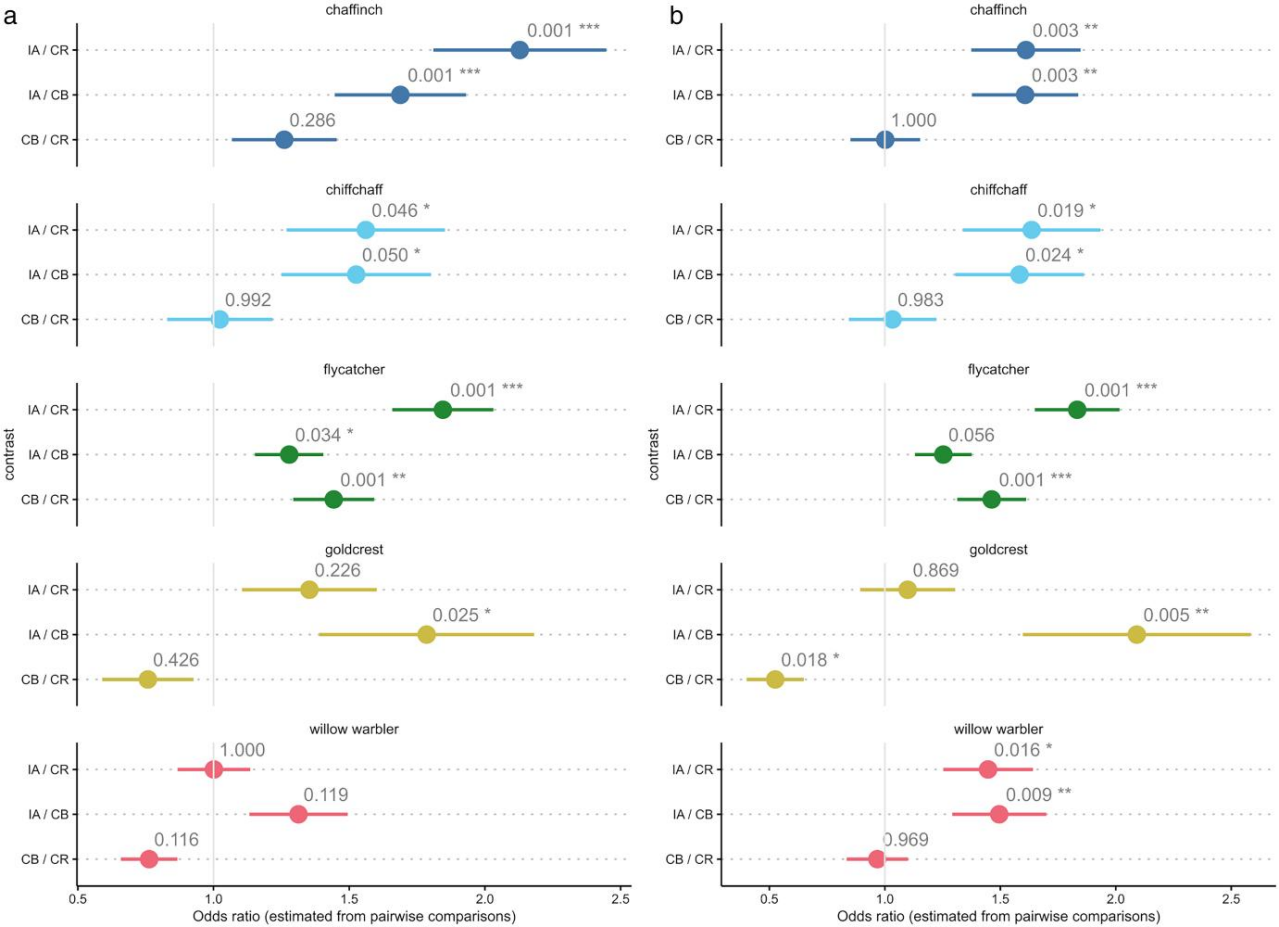
Pairwise comparisons of playback treatment effects on the probability of a) the bird's song overlapping in >50% of its duration with that of an acoustic competitor (sunbird, CR; illadopsis, IA; camaroptera, CB); b) a bird starting its song during the playback of a 5-s song of an acoustic competitor (sunbird, CR; illadopsis, IA; camaroptera, CB), shown as odds ratios with 95% confidence intervals. Odds ratios >1 indicate higher odds of a) bird overlapping or b) bird beginning the song during the competitor's song in the first playback treatment group contrast compared with second. A vertical line at 1 marks the null value (no difference between groups). *P*-values are Tukey-adjusted and annotated next to each point (*P < 0.05; **P < 0.01; ***P < 0.001).

Post-hoc comparisons indicated that common chaffinch songs overlapped significantly less with the songs of CR (*P* < 0.001) and CB (*P* < 0.001; Tables 4 and S2, Fig. 4a) than with songs of IA. There was no significant difference in the proportion of songs overlapping with CR compared with CB (*P* = 0.286, Table 4), and no significant effect of the experiment duration on the proportion of overlapping chaffinch songs (*P* = 0.785, Table S2).

**Table 4:** Pairwise comparisons of estimated marginal means (on the log-odds scale) for the probability of a bird overlapping its song by more than 50% with that of an acoustic competitor (sunbird, CR; illadopsis, IA; camaroptera, CB), based on a binomial generalized linear model.

	Contrast	Odds ratio	SE	*Z*-ratio	*P*-value
**Common chaffinch**	IA/CB	1.69	0.242	3.657	**<0**.**001**
	IA/CR	2.13	0.319	5.045	**<0**.**001**
	CB/CR	1.26	0.193	1.510	0.286
**Common chiffchaff**	IA/CB	1.53	0.275	2.344	0.050
	IA/CR	1.56	0.292	2.380	**0**.**046**
	CB/CR	1.02	0.194	0.121	0.992
**Pied flycatcher**	IA/CB	1.28	0.126	2.493	**0**.**034**
	IA/CR	1.85	0.187	6.058	**<0**.**001**
	CB/CR	1.44	0.149	3.549	**0**.**001**
**Goldcrest**	IA/CB	1.76	0.396	2.611	**0**.**025**
	IA/CR	1.35	0.248	1.648	0.226
	CB/CR	0.76	0.169	−1.246	0.426
**Willow warbler**	IA/CB	1.31	0.181	1.973	0.119
	IA/CR	1.00	0.134	0.011	0.999
	CB/CR	0.76	0.104	−1.983	0.116

Odds ratios >1 indicate higher odds of beginning singing in the first playback treatment group of the contrast. *P*-values are Tukey-adjusted for multiple comparisons. Full model output of the binomial logistic regressions is available in Table S2.

The common chiffchaff also overlapped significantly less with the songs of CR (*P* = 0.046) and CB (*P* = 0.050; Tables 4 and S2, Fig. 4a) than with songs of IA. There was no significant difference in the proportion of songs overlapping with CR compared with CB (*P* = 0.992, Table 4), but the birds overlapped with significantly fewer songs over the course of the experiment (OR = 0.97; 95% CI = 0.94, 0.99; *P* = 0.030, Table S2).

The pied flycatchers overlapped with significantly fewer songs of CR (*P* < 0.001) and CB (*P* = 0.034; Tables 4 and S2, Fig. 4a) than with songs of IA. Pairwise comparisons also revealed that the flycatcher overlapped significantly less with CR than with CB (*P* = 0.001, Table 4). Over the course of the experiment the probability of the flycatcher song overlapping with an acoustic competitor increased (OR = 1.03; 95% CI = 1.01, 1.05; *P* < 0.001, Table S2).

The goldcrests overlapped with significantly fewer with songs of CB (*P* = 0.025) than IA (Tables 4 and S2, Fig. 4a). However, there was no significant difference in the probability of goldcrest songs overlapping with the acoustic competitor between the playbacks of CR and IA (*P* = 0.226) and between the playbacks of CB and CR (*P* = 0.426, Table 4). Over the course of the experiment the probability of the goldcrest song overlapping with an acoustic competitor also increased (OR = 1.04; 95% CI = 1.00, 1.09; *P* = 0.042, Table S2).

Post-hoc pairwise comparisons with Tukey-adjusted *P*-values revealed no significant difference in the probability of song overlap of willow warblers and any of the 3 playback treatment pairs (Table 4, Fig. 4a). There was no significant effect of the experiment duration on the proportion of overlapping willow warbler songs (*P* = 0.141, Table S2).

### Song beginning

Post-hoc comparisons indicated that common chaffinches were significantly less likely to start their songs during the songs of CR (*P* = 0.003) and CB (*P* = 0.003; Tables 5 and S3, Fig. 4b) than with songs of IA. There was no significant difference in the proportion of songs beginning during the songs of CR compared with CB (*P* = 0.999, Table 5), and no significant effect of the experiment duration on the proportion of chaffinch songs beginning during the competitor's song (*P* = 0.381, Table S3).

**Table 5:** Pairwise comparisons of estimated marginal means (on the log-odds scale) for the probability of a bird beginning its song during a song of an acoustic competitor (sunbird, CR; illadopsis, IA; camaroptera, CB), based on a binomial generalized linear model.

	Contrast	Odds ratio	SE	*Z*-ratio	*P*-value
**Common chaffinch**	IA/CB	1.61	0.230	3.315	**0**.**003**
	IA/CR	1.61	0.237	3.240	**0**.**003**
	CB/CR	1.00	0.151	0.014	0.999
**Common chiffchaff**	IA/CB	1.58	0.278	2.613	**0**.**024**
	IA/CR	1.64	0.298	2.697	**0**.**019**
	CB/CR	1.03	0.189	0.178	0.983
**Pied flycatcher**	IA/CB	1.25	0.123	2.302	0.056
	IA/CR	1.83	0.184	6.052	**<0**.**001**
	CB/CR	1.46	0.149	3.727	**<0**.**001**
**Goldcrest**	IA/CB	2.091	0.493	3.126	**0**.**005**
	IA/CR	1.009	0.206	0.504	0.869
	CB/CR	0.526	0.124	−2.725	**0**.**018**
**Willow warbler**	IA/CB	1.495	0.204	2.955	**0**.**009**
	IA/CR	1.447	0.194	2.751	**0**.**016**
	CB/CR	0.968	0.133	−0.239	0.969

Odds ratios >1 indicate higher odds of beginning singing in the first playback treatment group of the contrast. *P*-values are Tukey-adjusted for multiple comparisons. Full model output of the binomial logistic regressions is available in Table S3.

A similar pattern was observed in the common chiffchaff. The birds were significantly less likely to start their songs during the songs of CR (*P* = 0.019) and CB (*P* = 0.024; Tables 5 and S3, Fig. 4b) than with songs of IA. There was no significant difference in the proportion of songs beginning during the songs of CR compared with CB (*P* = 0.983, Table 5), and no significant effect of the experiment duration on the proportion of chiffchaff songs beginning during the competitor's songs (*P* = 0.846, Table S3).

The pied flycatchers were significantly less likely to begin their songs during the songs of CR (*P* < 0.001; Table 5, Fig. 4b) than during the song of IA. The flycatchers were also significantly less likely to start their song during a song of CR compared with a song of CB (*P* < 0.001, Table 5). However, pairwise comparisons revealed no significant difference in the proportion of songs beginning during the songs of IA and CB (*P* = 0.056) Over the course of the experiment the probability of the flycatcher song beginning during the song of an acoustic competitor also increased (OR = 1.03; 95% CI = 1.01, 1.05; *P* = 0.002, Table S3).

Goldcrests were significantly less likely to begin their songs during the songs of CB (*P* = 0.005) than during the songs of IA (Tables 5 and S3, Fig. 4b). Pairwise comparisons also revealed that the goldcrest was significantly more likely to start its song during a song of CR compared with a song of CB (*P* = 0.018, Table 5). However, there was no significant difference in the probably of goldcrest starting its song during the song of CR and IA (*P* = 0.869). Over the course of the experiment the probability of the goldcrest song starting during the song of an acoustic competitor also increased (OR = 1.07; 95% CI = 1.03, 1.12; *P* < 0.001).

Willow warblers were significantly less likely to start their songs during the songs of CR (*P* = 0.016) and CB (*P* = 0.009; Tables 5 and S3, Fig. 4b) than with songs of IA. There was no significant difference in the probability of the willow warbler starting its song during the song of CR compared with CB (*P* = 0.969, Table 5), and no significant effect of the experiment duration on the proportion of overlapping chaffinch songs (*P* = 0.162, Table S3).

## Discussion

Our results support the temporal acoustic partitioning hypothesis, which predicts that animals will adjust the timing of their vocalizations to avoid spectral overlap with other sounds (Hödl 1977). We also demonstrate that songbirds inhabiting boreal forest have multiple different species-specific strategies for song masking avoidance, and can vary their behavior according to the frequency range and structure of the acoustic competitor songs.

All 5 of the study species avoided overlapping with and starting their songs during the broad-spectrum songs of camaroptera more than with songs of illadopsis whose narrow frequency spectrum song did not fully mask the study species' song. Additionally, 4 of the species (chaffinch, chiffchaff, pied flycatcher and willow warbler) also avoided starting their songs during the broad-spectrum sunbird songs. Both of these acoustic competitors have songs which fully overlapped with the frequency of the study species song. However, the sunbird songs continuously mask the full 5-s duration of the song, while in camaroptera songs include silent gaps between individual notes. Our results suggest that the rate of frequency range overlap, rather than the structure of the interfering song determine the temporal adjustment of the bird singing response, and are consistent with the previous studies demonstrating that birds can modify their song timing and sing in between the vocalizations of other species to avoid signal masking (eg Popp et al. 1985; Brumm 2006; Bleach et al. 2015; Herrick et al. 2018; Yang et al. 2014; Wilson et al. 2016).

The pied flycatcher was the only species in our study observed to modify its song rate in response to unfamiliar acoustic competitors. Song rate adjustment strategies in response to interfering sound can vary between species, ranging from birds reducing their song rate to avoid signal masking by similar sounds (Luther 2009; Budka et al. 2023), to individuals increasing their song rate (signal redundancy) to improve the chances of their signal reaching the receiver (Wiley and Richards 1982; Brumm and Slater 2006). The pied flycatchers sang fewer songs and were also less likely to overlap with both of the acoustic competitors masking their full frequency range (sunbird and camaroptera), than with illadopsis. However, in comparison to other species in our study, flycatchers consistently produced almost double the number of songs during each playback treatment. Each 150-s playback treatment consisted of alternating 5-s songs with 5-s silence. Thus, it is possible that the lower song rates during sunbird and camaroptera playbacks are the direct result of the flycatchers avoiding overlapping with and singing during the competitor's songs in a limited playback treatment session, rather than actively reducing the number of songs during a period of interfering sound.

In addition to song overlap avoidance, chiffchaffs, chaffinches and goldcrests modified the duration of their songs, shortening them in response to the 2 broad-frequency spectrum acoustic intruders. Shortening the songs could allow the bird to fit more songs into the temporal windows between vocalizations of acoustic competitors without having to reduce the number of songs produced. Although pied flycatcher songs can also vary in length, song duration is a signal of quality, and males who hold better territories typically sing longer and more complex songs (Lampe and Espmark 2003). This may explain why the flycatchers did not shorten their songs in response to acoustic competitors. In contrast, song frequency rather than song duration is correlated with male quality in chiffchaffs (Linhart et al. 2012) and many other passerines (Cardoso 2012). It is therefore possible that the differences in behavioral response to acoustic competition exhibited by different species are a result of not only their song frequency range and structure parameters, but also of the type of species-specific information these parameters encode.

In most of the study species we observed the significant effect of the experiment duration on the response to playback, with species changing their song rate, song duration or the level of overlap during the later phases of the experiment. Each experiment lasted 40 min, and consisted of 4 10-min sessions of the same sequence of 4 different playback treatments. This ensured that we recorded the responses of each study bird to each playback treatment at least once, as the birds could freely move in and out of range of our speakers. It is possible that over the course of the experiment the birds habituated to the novel sounds, and the changes in responses over time are the result of habituation.

To create novel acoustic competitors we modified the song frequency range of all playback sound samples to match the frequency spectrum of the target study species. As a result, the rapid series of notes in modified camaroptera songs resembled alarm calls of many passerines. Such calls are highly conserved between species (Dutour et al. 2017) and are used in interspecific communication, for example to elicit mobbing (Caro 2005). During our experiments we also observed multiple passerine species approach the speaker during the playback of camaroptera song, but did not observe such behavior in response to sunbird songs. This suggests that behavioral responses to camaroptera songs could also be motivated by aggression, rather than just acoustic overlap avoidance, particularly in the case of goldcrests, as this species did not show song overlap avoidance with sunbird songs. Thus, while our results suggest that the frequency range—rather than the structure—of the interfering song primarily drives the birds' temporal avoidance behavior, the information content of the interfering song may also influence their response. Future studies that incorporate varying levels of song structure complexity, as well as differences in the timing between interfering song syllables, could help clarify the extent to which structural complexity affects birds' behavioral responses to acoustic competition.

The frequency range of goldcrest songs is above other species examined in this study. Unlike in the case of tropical forest habitats, where birds share acoustic space with multiple species occupying high frequency spectrum range such as cicadas (Hart et al. 2015; Stanley et al. 2016; Berman et al. 2024), in a boreal forest the higher frequency spectrum band contains fewer natural broad-spectrum sounds that are likely to mask a goldcrest song. Planque and Slabbekoorn (2008) found evidence for acoustic overlap avoidance only in species occupying the most popular frequency spectrum bandwidth. Although goldcrests shortened their songs during the playbacks of both of the broad-frequency novel acoustic intruders, the lack of song overlap avoidance with sunbird could be the result of low number of naturally occurring acoustic competitors. This can be particularly important in light of climate change likely modifying the composition of future acoustic communities and exposing species to new acoustic competitors as a result of range expansions.

While our study demonstrates temporal avoidance strategies in the species examined, we did not assess alternative mechanisms birds may use to avoid competition. Specifically, due to interference from our playback stimuli, we were unable to measure the spectral frequencies of songs produced in response to the playbacks—particularly when they overlapped with the broadband songs of sunbirds. As a result, we could not evaluate spectral adaptation (ie altering song frequency to avoid acoustic masking) as a potential competition avoidance strategy (Lopez et al. 1988; Goodwin and Podos 2013). All 5 species in our study produce stereotyped songs, and although we did not observe clear shifts in song frequency during playback trials, it remains possible that birds could adjust their spectral output in response to overlapping sounds. Birds may also increase the amplitude of their songs to counteract the masking effects of competing signals (Brumm 2004; Kunc and Schmidt 2021). Although we did not measure song amplitude in our study, such adjustments could represent another competition avoidance strategy and may help explain interspecific differences in song overlap.

In our study we present experimental evidence for interspecific acoustic competition avoidance in all 5 boreal songbird species we tested. While the type and level of response varied between species, all birds showed higher temporal avoidance of acoustic competitors which could completely mask them on the frequency spectrum, than of an acoustic competitor which masked only part of their song frequency band. We also demonstrate that the behavioral response to acoustic interference can be species-specific, resulting in different metrics—such as song rate or song duration—revealing the behavioral strategies of different species.

## Supplementary Material

araf154_Supplementary_Data

## Data Availability

Analyses reported in this article can be reproduced using the data provided by Staniewicz et al. (2026).
