# Supplementary material for: Frequency masking drives species-specific temporal avoidance strategies in boreal songbirds
Source: Behav Ecol. 2025 Dec 22;37(2):araf154. doi: 10.1093/beheco/araf154 (PMC12835922; doi:10.1093/beheco/araf154)
Supplement: araf154_Supplementary_Data [file araf154_supplementary_data.zip › Table S1.docx]

Table S1: Numbers of playback treatment sequence combinations within the 10-minute session of the experiment presented to each species. Each playback code corresponds to the “Playback” in the data file, and where two playback codes correspond to the same playback sequence combination, different songs were used to create the two playback files.

| Playback code | Playback sequence combination | chaffinch | flycatcher | chiffchaff | willow warbler | goldcrest |
| --- | --- | --- | --- | --- | --- | --- |
| XX1 | silence-IA-CB-CR | 1 | 1 | 1 | 1 | 1 |
| XX27 | silence-CB-IA-CR | 1 | 1 | 1 | 1 | 1 |
| XX26 | silence-CR-IA-CB | 0 | 1 | 1 | 1 | 1 |
| XX22, XX24 | IA-silence-CB-CR | 2 | 2 | 2 | 2 | 2 |
| XX28 | IA-silence-CR-CB | 1 | 0 | 0 | 0 | 0 |
| XX4 | IA-CR-silence-CB | 1 | 1 | 1 | 1 | 1 |
| XX23 | IA-CR-CB-silence | 1 | 1 | 1 | 1 | 1 |
| XX5 | IA-CB-CR-silence | 0 | 1 | 0 | 0 | 0 |
| XX25 | CB-silence-IA-CR | 2 | 1 | 1 | 0 | 1 |
| XX29 | CB-CR-IA-silence | 0 | 0 | 1 | 1 | 0 |
| XX6 | CB-CR-silence-IA | 0 | 0 | 0 | 0 | 1 |
| XX2, XX3 | CR-silence-CB-IA | 2 | 2 | 2 | 3 | 2 |
| XX21 | CR-CB-IA-silence | 1 | 1 | 1 | 1 | 1 |
