# Supplementary material for: Frequency masking drives species-specific temporal avoidance strategies in boreal songbirds
Source: Behav Ecol. 2025 Dec 22;37(2):araf154. doi: 10.1093/beheco/araf154 (PMC12835922; doi:10.1093/beheco/araf154)
Supplement: araf154_Supplementary_Data [file araf154_supplementary_data.zip › Table S2.docx]

**Table S2:** Results of the binomial logistic regressions comparing the probability of a bird overlapping >50% of duration of its song between the three acoustic competitors (sunbird, CR; illadopsis, IA; camaroptera, CB).

|  | **Coefficients** | **Estimate** | **SE** | **Z** | **P** |
| --- | --- | --- | --- | --- | --- |
| Common chaffinch | (Intercept) IA | 0.094 | 0.150 | 0.629 | 0.529 |
|  | CB | -0.524 | 0.143 | -3.657 | **<0.001** |
|  | CR | -0.756 | 0.150 | -5.045 | **<0.001** |
|  | Sequence | -0.004 | 0.014 | -0.273 | 0.785 |
| Common chiffchaff | (Intercept) IA | -0.032 | 0.166 | -0.195 | 0.845 |
|  | CB | -0.422 | 0.180 | -2.344 | **0.019** |
|  | CR | -0.445 | 0.187 | -2.380 | **0.017** |
|  | Sequence | -0.035 | 0.016 | -2.170 | **0.030** |
| Pied flycatcher | (Intercept) IA | -0.451 | 0.104 | -4.347 | **<0.001** |
|  | CB | -0.246 | 0.099 | -2.493 | **0.013** |
|  | CR | -0.612 | 0.101 | -6.058 | **<0.001** |
|  | Sequence | 0.031 | 0.009 | 3.351 | **<0.001** |
| Goldcrest | (Intercept) IA | -0.433 | 0.193 | -2.238 | **0.025** |
|  | CB | -0.580 | 0.222 | -2.611 | **0.009** |
|  | CR | -0.306 | 0.184 | -1.648 | 0.099 |
|  | Sequence | 0.042 | 0.020 | 2.033 | **0.042** |
| Willow warbler | (Intercept) IA | -0.333 | 0.133 | -2.502 | **0.012** |
|  | CB | -0.272 | 0.138 | 11.973 | **0.049** |
|  | CR | -0.001 | 0.134 | -0.011 | 0.992 |
|  | Sequence | -0.018 | 0.012 | -1.472 | 0.141 |
