# Supplementary material for: Frequency masking drives species-specific temporal avoidance strategies in boreal songbirds
Source: Behav Ecol. 2025 Dec 22;37(2):araf154. doi: 10.1093/beheco/araf154 (PMC12835922; doi:10.1093/beheco/araf154)
Supplement: araf154_Supplementary_Data [file araf154_supplementary_data.zip › Table S3.docx]

**Table S3:** Results of the binomial logistic regressions comparing the probability of a bird beginning its song during a song of an acoustic competitor. Comparison between the three acoustic competitors (sunbird, CR; illadopsis, IA; camaroptera, CB).

|  | **Coefficients** | **Estimate** | **SE** | **Z** | **P** |
| --- | --- | --- | --- | --- | --- |
| Common chaffinch | (Intercept) IA | -0.083 | 0.149 | -0.558 | 0.577 |
|  | CB | -0.475 | 0.143 | -3.315 | **<0.001** |
|  | CR | -0.477 | 0.147 | -3.240 | **<0.001** |
|  | Sequence | 0.012 | .013 | 0.877 | 0.381 |
| Common chiffchaff | (Intercept) IA | 0.028 | 0.163 | 0.173 | 0.863 |
|  | CB | -0.459 | 0.176 | -2.613 | **0.009** |
|  | CR | -0.492 | 0.182 | -2.697 | **0.007** |
|  | Sequence | -0.003 | 0.015 | -0.193 | 0.846 |
| Pied flycatcher | (Intercept) IA | -0.345 | 0.103 | -3.362 | **<0.001** |
|  | CB | -0.226 | 0.098 | 2.302 | **0.021** |
|  | CR | -0.606 | 0.100 | -6.052 | **<0.001** |
|  | Sequence | 0.027 | 0.009 | 3.032 | **0.002** |
| Goldcrest | (Intercept) IA | -0.865 | 0.201 | -4.302 | **<0.001** |
|  | CB | -0.738 | 0.236 | -3.136 | **0.002** |
|  | CR | -0.094 | 0.187 | -0.504 | 0.614 |
|  | Sequence | 0.068 | 0.021 | 3.230 | **0.001** |
| Willow warbler | (Intercept) IA | -0.152 | 0.132 | -1.149 | 0.251 |
|  | CB | -0.402 | 0.136 | -2.955 | **0.003** |
|  | CR | -0.370 | 0.134 | -2.751 | **0.006** |
|  | Sequence | -0.017 | 0.012 | -1.398 | 0.162 |
